# Genome-wide identification and expression analysis of the xyloglucan endotransglucosylase/hydrolase gene family in poplar

**DOI:** 10.1186/s12864-021-08134-8

**Published:** 2021-11-08

**Authors:** Zihan Cheng, Xuemei Zhang, Wenjing Yao, Yuan Gao, Kai Zhao, Qing Guo, Boru Zhou, Tingbo Jiang

**Affiliations:** 1 State Key Laboratory of Tree Genetics and Breeding, Northeast Forestry University, Harbin, China; 2 Bamboo Research Institute, Nanjing Forestry University, 159 Longpan Road, Nanjing, 210037 China

**Keywords:** Poplar, XTH, Expression patterns, Salt stress

## Abstract

**Background:**

Xyloglucan endotransglucosylase/hydrolase (XTH) family plays an important role in cell wall reconstruction and stress resistance in plants. However, the detailed characteristics of XTH family genes and their expression pattern under salt stress have not been reported in poplar.

**Results:**

In this study, a total of 43 *PtrXTH* genes were identified from *Populus simonii* × *Populus nigra*, and most of them contain two conserved structures (Glyco_hydro_16 and XET_C domain). The promoters of the *PtrXTH* genes contain mutiple cis-acting elements related to growth and development and stress responses. Collinearity analysis revealed that the XTH genes from poplar has an evolutionary relationship with other six species, including *Eucalyptus robusta*, *Solanum lycopersicum*, *Glycine max*, *Arabidopsis*, *Zea mays* and *Oryza sativa*. Based on RNA-Seq analysis, the *PtrXTH* genes have different expression patterns in the roots, stems and leaves, and many of them are highly expressed in the roots. In addition, there are11 differentially expressed *PtrXTH* genes in the roots, 9 in the stems, and 7 in the leaves under salt stress. In addition, the accuracy of RNA-Seq results was verified by RT-qPCR.

**Conclusion:**

All the results indicated that XTH family genes may play an important role in tissue specificity and salt stress response. This study will lay a theoretical foundation for further study on molecular function of XTH genes in poplar.

**Supplementary Information:**

The online version contains supplementary material available at 10.1186/s12864-021-08134-8.

## Background

As the external supporting structure of cells, cell wall largely determines the shape and size of cells in the process of plant growth and development. The main components of plant cell wall are cellulose, lignin, hemicellulose and pectin [1]. Xyloglucan is the most important hemicellulose in the primary cell wall of dicotyledons [2]. Xyloglucan endotransglucosylase/hydrolase (XTH) is widely present in plant cells, which can catalyze the cleavage and polymerization of xyloglucan molecules, and achieve cell remodeling by regulating the elasticity and ductility of cells [3]. It is a key enzyme in the process of cell wall reconstruction in plants.

XTH family genes contain a typical catalyze enzymatic reaction motif (HDEIDFEFLG), in which the first glutamic acid residue (E) is affinity site and the second is proton donor [4, 5]. XTHs can be used to form a covalent glycosylase intermediate, which can be decomposed by water to carry out hydrolysis reaction, or enter the sugar substrate to produce transglycosylation [3]. According to the conserved motifs, the XTH family can be divided into three groups (I, II, and III), and the group III is divided into IIIA and IIIB group [6]. It has been found that the XTHs in groups I, II and IIIB have significant xyloglucan endohydrolase (XET) activity, while the XTHs in IIIA show xyloglucan endoglucosidase (XEH) activity [7]. Moreover, a small part of outlier group (early diverting) was found in detailed clade [7, 8]. In summary, XTH family is divided into I / II, IIIA, IIIB and the early diverting groups [9].

The expression characteristics of many XTH family genes are different in different tissues during different developmental stages. For example, poplar *PtxtXET16–34* was specifically expressed in the developing wood, and overexpression of the gene could increase xyloglucan content in the xylem of the primary wall and promote the growth of vessel element [10]. In Arabidopsis, *AtXTH17*, *AtXTH18*, *AtXTH19* and *AtXTH20* were specifically expressed in roots and play an important role in root elongation and root hair initiation [11, 12]. *AtXTH21* participates in the growth of primary roots by changing the cellulose deposition and the extensibility in the cell wall [13]. *AtXTH31* can encode genes of root extension region expression and participates in root elongation and growth [14]. In addition, some XTH genes are active in the development of leaf [15], fruit [16] and flower [17]. Many studies have also indicated that the expression of XTHs was regulated by plant hormones. For example, the expression of rice *OsXTH8* [18] and Arabidopsis *AtXTH21* [13] was up-regulated by gibberellin treatment. The expression of banana *MA-XET1* was induced by ethylene and participates in the ripening and softening of peel and pulp [19]. Under the treatment of brassinosteroid (BR), the expression of *AtXTH22* and *AtXTH24* genes increased significantly in Arabidopsis, thereby promoting the elongation of cell walls [20].

In particular, XTH genes play an important role in abiotic stress in plants. The content of xyloglucan was reduced in *AtXTH31* mutant of *Arabidopsis*, which leads to a decrease in the content of absorbed Al^3+^, thereby improving the aluminum stress tolerance [14]. Similarly, Arabidopsis *xth17* and *xth15* mutants had higher aluminum tolerance compared with wild-type plants [21, 22]. *CaXTH3* gene in pepper can be induced by drought, high salt and cold, and overexpression of the gene leads to severe fold of Arabidopsis leaves, however, which improves the drought and salt tolerance of transgenic plants [23]. Under low temperature, the expression of persimmon *DkXTH6* gene was inhibited, while *DkXTH7* showed a high level of transcription [24]. Overexpression of *DkXTH1* enhanced the tolerance of transgenic Arabidopsis to salt, ABA and drought stress, and affected the growth of roots and leaves [25].

The first XTH gene was discovered in cowpea [26], then the XTH family genes were established in other species. Among them, a total of 33, 29, 35, and 25 XTH genes were identified in Arabidopsis [11], rice [27], sorghum [28], and tomato [16], respectively. In poplar, Geisler-Lee [29] found 41 XTH genes, and Ye [30] found 42 genes. However, they both did not systematically analyze their expression pattern under salt stress in poplar. In the present research, we found 43 XTHs family genes from poplar. And we determined the evolutionary relationship of poplar XTH family by the analysis of gene structure and homologous collinearity. Meanwhile, the gene expression pattern of XTH family genes was profiled among different tissues under salt stress. This study provides a theoretical foundation for the research on salt-tolerance improvement of poplar plants.

## Result

### Phylogenetic tree analysis of XTH family

**Fig. 1:**
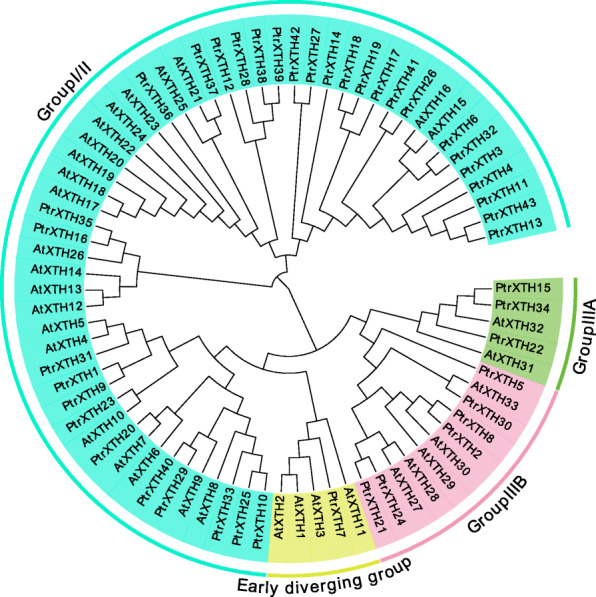
Phylogenetic analysis of the XTH proteins from poplar and Arabidopsis. The phylogenetic tree was constructed by MEGA6 software, with neighbor-joining method. The evolutionary tree was divided to 4 groups, each color represents one group

**Fig. 2:**
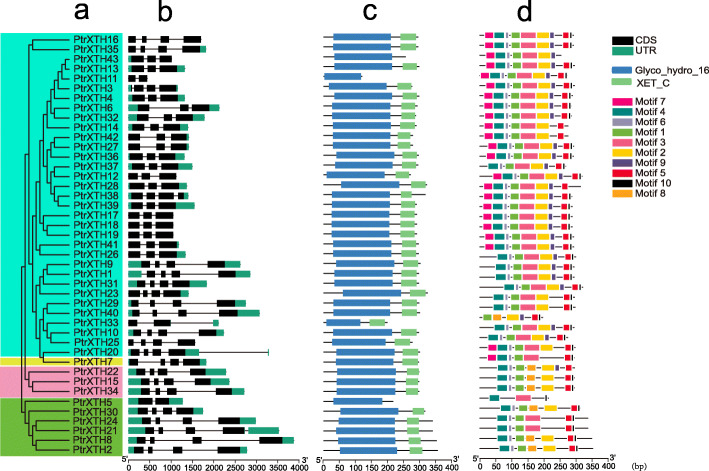
Characterization of the genes structure, conserved protein domain and motif of the poplar XTH genes. (**a**) Phylogenetic tree of poplar XTH genes family, (**b**) Gene structures, (**c**) Conserved protein domain, (**d**) Protein conserved motifs

In this study, 43 XTH members were found in poplar genome and named as *PtrXTH1* - *PtrXTH43* according to their position in the chromosome. The phylogenetic tree of XTH family genes from poplar and Arabidopsis was constructed by MEGA6. As shown in the Fig. 1, the XTH family could be divided into 4 groups (GroupI/II, Group IIIA, Group IIIB and Early diverging group), which was similar to that in *Brassica rapa* [9]. And the GroupI/IIcontained the most members with 33 members. Group IIIA had 6 members, Group 3B had 3 members, and group 4 had only one member (Fig. 2a).

The physicochemical properties of XTH proteins were also analyzed (Supplementary Table 1). PthXTH2 encoded the largest amino acids (351) with the molecular weight (MW) of 40.77 kDa, while PthXTH11 had the smallest amino acids (117) with the MW of 13.44 kDa. The theoretical isoelectric point (PI) ranged from 4.53 to 9.67, while the instability coefficient ranged from 27.44 to 55.83. In addition, only one protein had grand average of hydropathicity (GRAVY) greater than 0, which was regarded as hydrophobic protein, and the others were less than 0, which were expressed as hydrophilic protein.

**Fig. 3:**
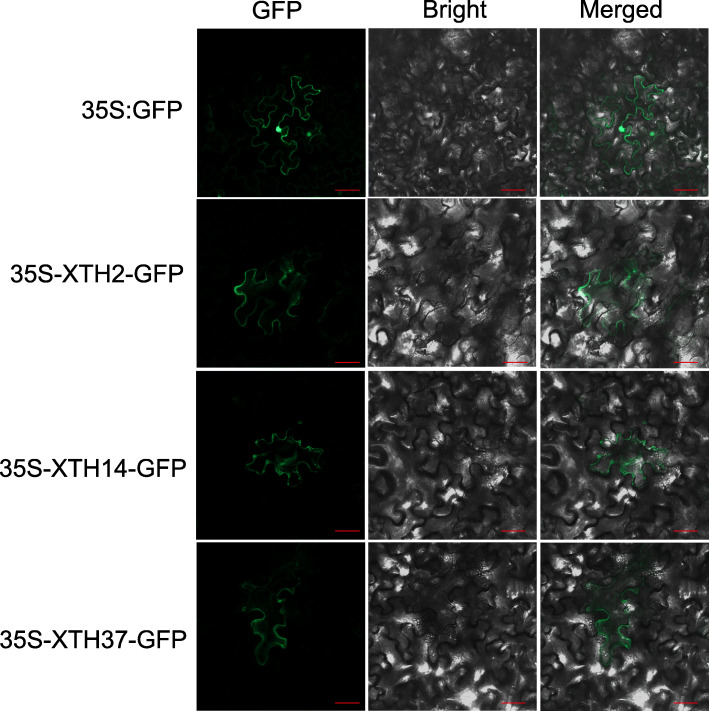
Subcellular localization of PtrXTH2, PtrXTH14 and PtrXTH37 proteins. The control (35S-GFP) and fusion vector (35S-XTH2-GFP, 35S-XTH14-GFP, 35S-XTH37-GFP) were separately transiently expressed in *N. benthamiana* leaves by agrobacterium-mediated method. Scale bar = 20 um

For subcellular localization, there were 8 genes predicted to be located in the extracellular, and the other 35 proteins in the plasma membrane, and 80% of XTH proteins had signal peptides. Then we randomly selected *PtrXTH2*, *PtrXTH14* and *PtrXTH37* genes to verify the predicted results (Fig. 3). The signal of positive control (35S-GFP) was detected in the *Nicotiana benthamiana* leaves nucleus and plasma membrane, while 35S-XTH2-GFP, 35S-XTH14-GFP and 35S-XTH37-GFP were detected in the plasma membrane. The results verified the accuracy of the subcellular localization prediction of XTH proteins.

### Gene structure analysis of poplar XTH family genes

In general, the genes that are clustered in one group have a similar structure. In this study, the genes in GroupI/IIhave 2 to 3 introns except *PtrXTH11* (1 intron) and *PtrXTH1* (4 introns), and the genes in Group IIIA have 3 introns except *PtrXTH5* (1 intron). In addition, there were 3 introns in the genes in Group IIIB and Early diverging group, respectively (Fig. 2b). We used PFAM data to analyze the conserved domain of the 43 XTHs. Only three proteins including *PtrXTH11*, *PtrXTH43* and *PtrXTH5* had one conserved domain (Glyco_hydro_16), while the other 40 genes had both Glyco_hydro_16 and XET_C domain (Fig. 2c).

In addition, we found 10 related motifs through MEME online software (Fig. 2d and Supplementary Table 2). The longest one contained 50 amino acids (motif 3 and motif 4), and the shortest contained 7 amino acids (motif 7) (Fig. 2d). Glyco_hydro_16 contained the motifs 1, 4, 6, 7 and 8, but not all protein had the 5 motifs. For instance, XET_C contained the motif 5 and motif 10. The results were similar to those in the previous study [9].

### Cis-acting elements in *PtrXTHs*

**Fig. 4:**
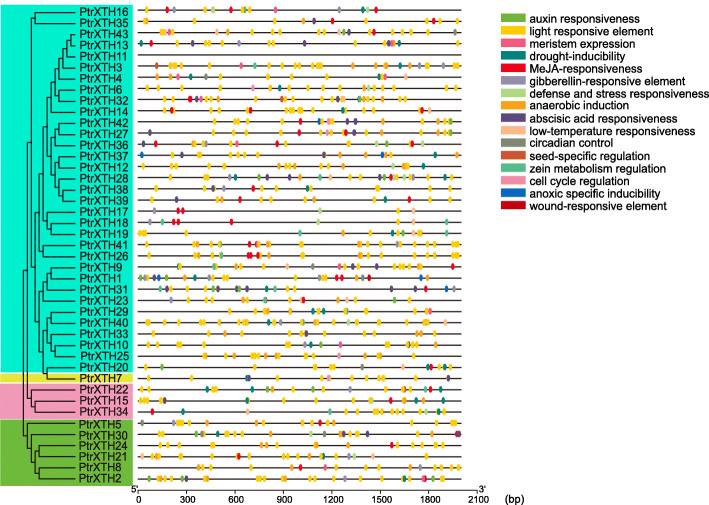
Cis-elements analysis of the poplar XTH gene promoters. The left side represents the phylogenetic tree of the poplar XTH genes, and the rights with different colored ellipses represent different elements

In this study, the 2000 bp upsteam sequences of the 43 *PtrXTHs* were analysed by PlantCARE. As shown in Fig. 4 and Supplementary Table 3, we found 16 types of cis-elements in the upstream promoter sequences of *PtrXTHs.* Among them, there were many elements related to abiotic stresses such as drought-inducibility, defense and stress responsiveness, low-temperature responsiveness, and wound-response. The elements including meristem expression, analytical induction, circadian control, seed-specific regulation, cell cycle regulation and light responsive were related to plant growth and development. The elements such as auxin responsiveness, MeJA-responsiveness, gibberellin-responsive, abscisic acid responsiveness and zeatin metabolism regulation were related to hormones response. In addition, there were multiple elements in response to a certain stress (Supplementary Table 3). For example, I-box, AE-box, and G-Box were involved in light responsive. P-box, TATC-box and GARE-motif were involved in gibberellin-responsive. The results showed that many *PtrXTHs* were involved in plant growth and stress response.

### Chromosomal distribution and synteny analysis of *PtrXTHs*

**Fig. 5:**
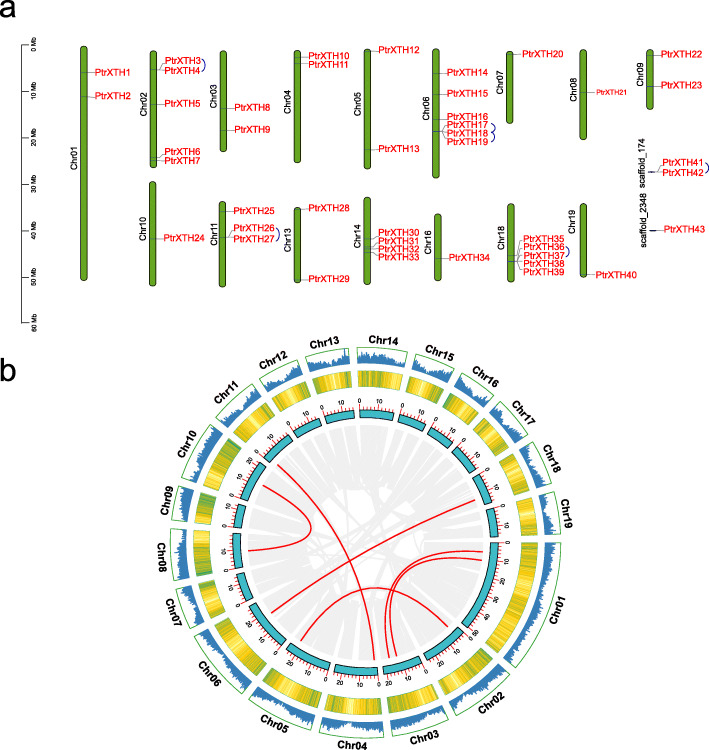
Gene location and repetitive events of the poplar XTH genes. (**a**) Chromosome location information on the of the poplar XTH genes. The blue arcs represent different pairs of tandem repeat genes. (**b**) Fragment duplication analysis of the poplar XTH genes. The line and heat map in the outer circle represent gene density on the chromosome, the red lines represents the fragment duplicate gene pair, and the gray lines represent the synteny blocks of the XTH genes in poplar genome

We mapped the poplar XTH family genes to poplar chromosomes through genome annotation. As shown in Fig. 5a, the 43 *PtrXTHs* were not centrally allocated to a certain chromosomes, while they were randomly allocated to as many chromosomes as possible in a scattered manner. For example, there was only one XTH gene allocated on chromosomes 7, 8, 10, 16, 19, and two on the chromosomes 1, 3, 4, 5, 9 and 13, and six on the chromosome 6, which had the most members. Interestingly, there was no *PtrXTHs* on the chromosomes 12, 15, and 17.

We analyzed the repetitive events of the *PtrXTHs* through MCscan. As shown in Fig. 5a and Supplementary Table 4, there were 6 pairs of tandem repetitive events among 43 *PtrXTHs*, of which *PtrXTH18* shared two pairs of repetitive events. And 6 pairs of fragment duplication events were also identified in *PtrXTHs* (Fig. 5b), which were distributed on the different chromosomes.

**Fig. 6:**
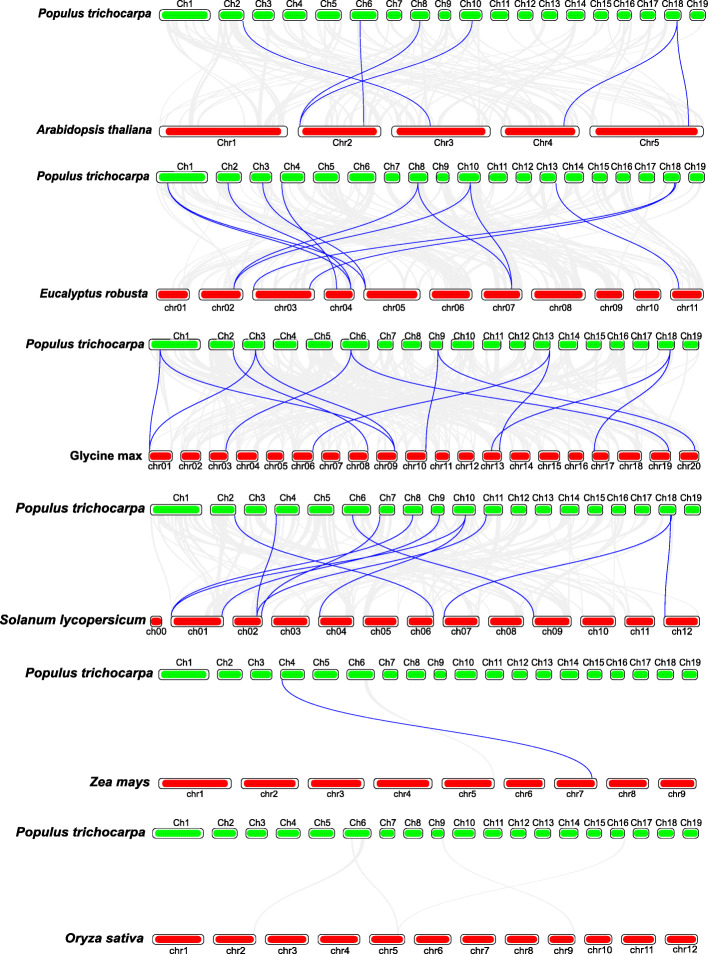
Collinearity analysis of the XTH genes from poplar and other six species. The blue lines represent XTH syntenic gene pairs between poplar and other species, and the gray lines represent orthologous genesof poplar and other six species

In order to further study the evolutionary relationship of XTH family, we constructed systematic maps of the XTH genes between poplar and six other species, including four dicotyledonous (*E. robusta*, *S. lycopersicum*, *G. max* and *Arabidopsis*) and two monocotyledons (*Z. mays* and *O. sativa*). As shown in Fig. 6 and Supplementary Table 5, there were 8 homologous pairs in *Arabidopsis*, 14 pairs in *G. max*, 12 pairs in *E. robusta*, 11 pairs in *S. lycopersicum*, 1 pair in *Z. mays*, and 0 pairs in *O. sativa*. Among them, a total of 16 *PtrXTHs* showed high collinearity relationship with other 6 species (7 in *Arabidopsis*, 8 in *G. max*, 9 in *E. robusta*, 10 in *S. lycopersicum*, and 1 in *Z. mays*). In addition, the *PtrXTHs* shared homologous pairs in the different species (Fig. S1). For example, *PtrXTH10* was homologous with the gene from *E. robusta*, *S. lycopersicum* and *Z. mays*, and *PtrXTH7*, *PtrXTH15* and *PtrXTH37* were shared in *G. max*, Arabidopsis and *S. lycopersicum*.

### Expression patterns of *PtrXTH* genes in different tissues

**Fig. 7:**
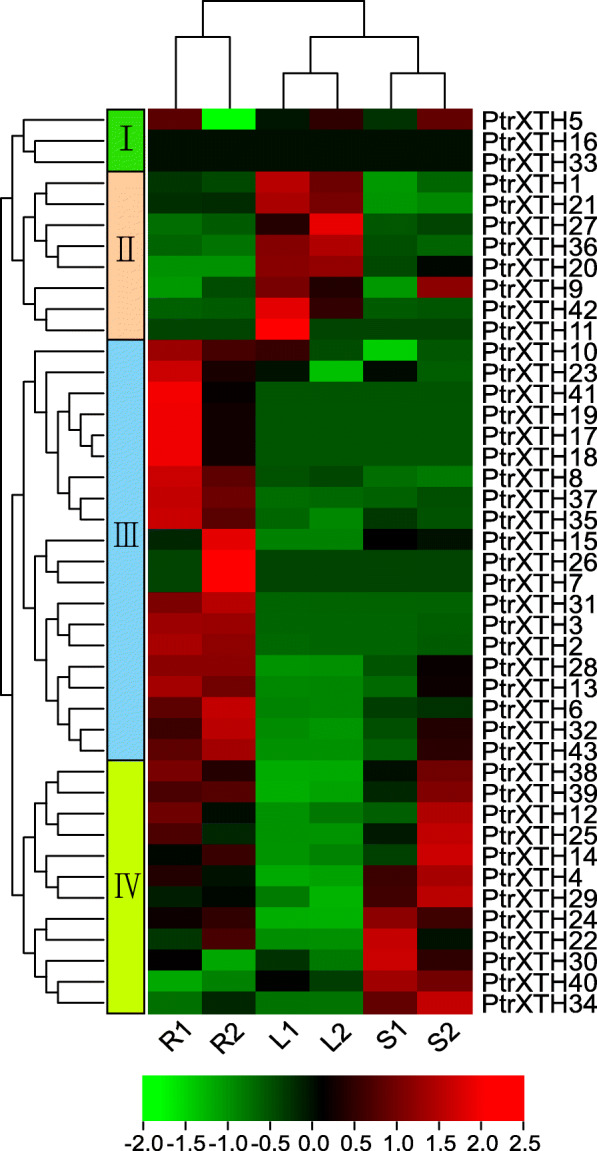
Expression pattern of the poplar XTH genes in different tissues. Red represents high expression, green represents low expression. The left represents four gene clusters, R, L, and S represents roots, leaves and stems without salt stress, respectively

In order to explore the expression patterns of *P. simonii × P. nigra* XTH genes, the expression level of *PtrXTHs* in the different tissues was profiled by RNA-Seq. As shown in Fig. 7 and Supplementary Table 6, the XTH genes were divided into 4 groups according to their expression patterns. Among them, the genes in the group 2, group 3 and group 4 were mainly expressed in the leaves, roots and stems, respectively. However, the expression of the genes in the group 1 was mixed. Similarly, some duplicate gene pairs had similar expression patterns. For example, *PtrXTH1* and *PtrXTH9* were expressed in the leaves, and *PtrXTH17* and *PtrXTH18* were expressed in the roots. However, some tandem gene pairs showed different expression trends. For instance, the *PtrXTH21* was expressed in the leaves, while the *PtrXTH24* gene was mainly expressed in the stems. The *PtrXTH26* gene was mainly expressed in the roots, while the *PtrXTH27* gene was mainly expressed in the leaves.

### *PtrXTHs* expression analysis under salt stress

**Fig. 8:**
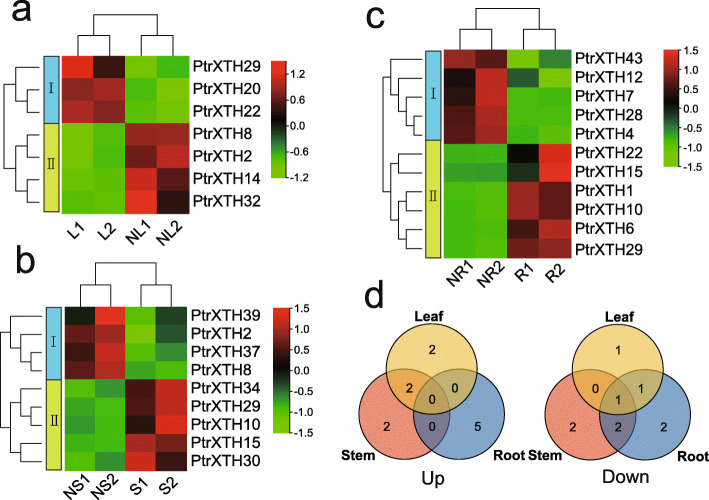
Expression pattern of *PtrXTH* genes under salt stress. (**a**-**c**) Heatmaps of DEGs in leaves, stems and roots, respectivley. R, L, and S represents the roots, leaves and stems without salt stress, respectively. NR, NL, and NS represents the roots, leaves and stems under salt stress, respectively. (**d**) Venn diagrams of DEGs among the three tissues

We also analyzed gene expression patterns of *PtrXTHs* in different tissues under salt stress by RNA-Seq. As shown in Fig. 8 and Supplementary Table 7, there were 7 differential expression genes (DEGs) in the leaves (4 up- and 3 down-regulated), 9 in the stems (4 up- and 5 down-regulated), and 11 in the roots (5 up- and 6 down-regulated). In addition, we analyzed the shared genes among different tissues. As shown in Fig. 8d, there were 3 shared genes (0 up- and 3 down-regulated) in the roots and stems,3 shared genes (2 up- and 1 down-regulated) in the stems and leaves, and 2 shared genes (0 up- and 2 down-regulated) in the roots and leaves. Furthermore, only one shared genes (0 up- and 1 down-regulated) were shared in the three tissues.

**Fig. 9:**
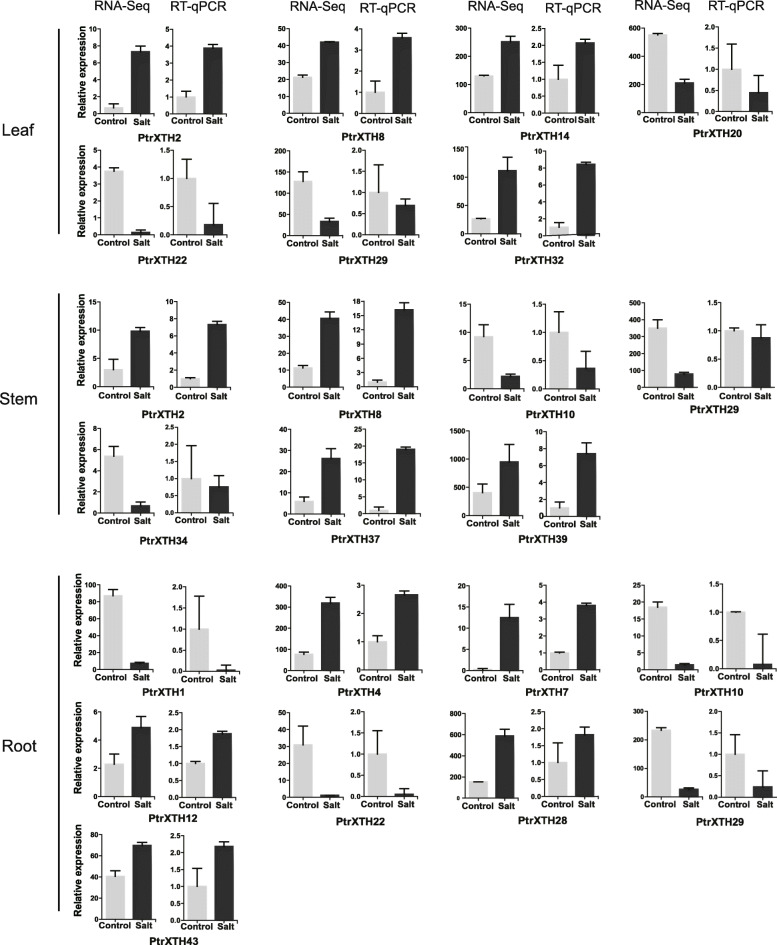
Gene expression levels in the three tissues by RNA-Seq and RT-qPCR. Salt and control represent with and without salt stress, respectively

To verify the accuracy of RNA-Seq data, the DEGs in the roots, stems and leaves were selected for RT-qPCR. As showed in Fig. 9, the relative expression levels of DEGs verified by RT-qPCR were in consistent with the mRNA abundance profiled by RNA-Seq analysis generally.

## Discussion

XTH family genes play an indispensable role in plants growth and development. In this study, we found 43 XTH genes from *P. trichocarpa*, which were named as *PtrXTH1* to *PtrXTH*43 according to their location on the chromosomes. The 43 *PtrXTHs* were divided into 4 subgroups compared with Arabidopsis, which was similar with rice and soybean. Most XTH genes contain two main conserved domains (Glyco_hydro_16 and XET_C domain). However, we found that *PtrXTH5* and *PtrXTH11* were lack of the XET_C domain. We speculate that there was a loss of XET_C domain during the evolution of the poplar XTH genes. In addition, we predicted that most PtrXTH proteins are located in the plasma membrane, while a small are located in the extracellular, which similar with the tobacco [31]. In our study, the homeopathic transformation experiment of tobacco found that the PtrXTH2, PtrXTH14 and PtrXTH37 proteins are located in the plasma membrane, and possibly in the cell wall. In addition, we found that Arabidopsis AtXTH30 (a homologous protein of PtrXTH2) protein and soybeans GmXTH23 (a homologous protein of PtrXTH14 and PtrXTH37) protein are located in the plasma membrane [32, 33], from which we speculate that they are localized in the plasma membrane.

Plants have experienced many whole-genome replication events in the process of evolution, including tandem repeat, fragment repeat and conversion events [34, 35]. In this study, we analyzed the collinearity events of XTH family genes in poplar. Interestingly, we found that the number of tandem repeat gene pairs was same to that of fragment repeat gene pairs. We speculate that the two evolutionary mechanisms were co-regulated in the process of evolution. In addition, we found that 7 out of 12 repetitive gene pairs had different expression patterns. For example, the fragment repetitive gene pair *PtrXTH10* / *PtrXTH25* had same structure and conserved motif, however, they displayed obviously different expression patterns. *PtrXTH10* was mainly expressed in the roots, while *PtrXTH25* was mainly expressed in the leaves. Therefore, we speculate that *PtrXTH* genes participate in the replication process, which causes gene mutations, leading to the change of gene function and expression patterns. This phenomenon is also occurred in the ERF family [36] and NAC family [37]. In addition, the collinear relationship between *PtrXTHs* and other six species were analyzed. We found that *PtrXTHs* had more collinear gene pairs with dicotyledons plants than that with monocotyledons plants.

XTH family genes are involved in the development of plant tissues and organs, and they have expression specificity of tissue and cell. In tobacco, *NtXTH4*, *NtXTH5*, *NtXTH12*, and *NtXTH19* had high expression levels in 19 tissues [31], and a similar situation occurred in barley [38]. In our study, we found that the *PtrXTHs* in groupI such as *PtrXTH16* and *PtrXTH31* were not expressed in the leaves, roots or stems, which may function in other organizations. The other three groups of *PtrXTH*s were expressed in the different tissues. However, there were more *PtrXTH*s expressed in the roots and stems than that in the leaves, which indirectly proves that *PtrXTHs* may play an important role in the development of root and stem. The previous studies have proved it. For example, Arabidopsis *XTH19* and *XTH23* (a homologous gene of *PtrXTH19*) were regulated by *BES1* to participate in the development of lateral roots through brassinosteroid signaling pathway, contributing to salt adaptation of lateral roots [39]. The deficiency of *XTH9* (a homologous gene of *PtrXTH40*) in Arabidopsis regulated the secondary wall thickening by triggering integrity signal of cell wall and stimulating the production of xylem cells [40].

During the process of growth and development, plants are subject to multiple abiotic stresses such as high temperature, salinity and drought [41]. XTH genes play an important role in abiotic stress. In our study, we analyzed the expression pattern of poplar XTH family genes under salt stress by RNA-Seq. We found 11 differential expression genes in the roots, 7 in the leaves and 9 in the stems. In addition, RT-qPCR was used to verify the accuracy of RNA-Seq analysis, which indicated the results were consistent in general. Phylogenetic analysis also showed that those genes play an important role in salt stress. For example, Arabidopsis gene *XTH30*, a homologous gene of *PtrXTH2* and *PtrXTH8*, was up-regulated under salt stress, and the lack of the gene slowed down the decrease of crystalline cellulose content and microtubule depolymerization under salt stress, which had a negative impact on salt tolerance [32]. Overexpression of *PeXTH* from *Populus euphratica* (a homologous gene of *PtrXTH28*) could increase water holding capacity and reduce salt concentration in fleshy tissues and mesophyll cells, which improved salt tolerance of transgenic tobacco [42]. All evidences indicate that *PtrXTH* genes play an important role in regulating stress response in plants.

## Conclusion

In this study, a total of 43 XTH genes were identified from poplar, which were divided into 4 groups. These genes were randomly distributed on the 16 chromosomes and 2 scaffolds in poplar. And as many as 12 pairs of duplication events among poplar XTH family genes were found, including 6 pairs of fragment duplication and 6 pairs of tandem duplication. In addition, we analyzed the homologous evolutionary relationship of XTH genes between poplar and other six species, which indicates the *PtrXTH*s had stronger homologous relationship with dicotyledonous plants, compared to monocotyledonous plants. Furthermore, we profiled the expression pattern of *PtrXTH*s in different tissues under salt stress through RNA-Seq and RT-qPCR. There were more *PtrXTH*s expressed in the roots and stems than that in the leaves, which indicates *PtrXTHs* may play an important role in the development of root and stem. All the results in this study provide a theoretical basis for function identification of poplar XTH genes.

## Materials and methods

### Identification of poplar XTH genes

All amino acid sequences of poplar XTH proteins were obtained from the genome of *P. trichocarpa v3.0* in Phytozome12 (https://phytozome.jgi.doe.gov/pz/portal.html). The hidden markov models of two typical XTH family protein structures (PF00722 and PF06955) were downloaded from Pfam database (http://pfam.xfam.org/). The HMMER3.0 [43] and SMART database (http://smart.embl-heidelberg.de/) were used to search and filter all potential XTH proteins in poplar. The ExPASy website (http://web.expasy.org/protparam/) was used to calculate physical and chemical parameters of the XTH proteins. SignalP v4.1 server (http://www.cbs.dtu.dk/services/SignalP/) was used to predict the signal peptide cleavage site, and ProtComp 9.0 (http://linux1.softberry.com) was used to predict the subcellular localization of the XTH proteins.

### Subcellular localization of PtrXTH2, PtrXTH14 and PtrXTH37 proteins

The full-length coding sequences of the *PtrXTH2, PtrXTH14* and *PtrXTH37* genes without stop codon were fused with pBI-121-GFP vector driven by 35S promoter, respectively. The fusion vector was transferred into GV3101 agrobacterium by liquid nitrogen freeze-thaw method. One-month-old *N. benthamiana* seedlings were used for transient transformation. The agrobacterium containing the fusion vector (35S-XTH2-GFP, 35S-XTH14-GFP, 35S-XTH37-GFP and 35S-GFP) was injected into the tobacco leaves with 1 ml syringes. Incubate in the dark for 36 h and observe tobacco cells under LMS800 laser confocal microscope.

### Phylogenetic analyses of *PtrXTH* proteins

The protein sequences of XTH family from poplar and *Arabidopsis* were downloaded from the Phytozome12 (https://phytozome.jgi.doe.gov/pz/portal.html) and TAIR online websites (https://www.arabidopsis.org/), respectively. ClustalW [44] was used for multiple sequence alignment of the proteins, and MEGA6 (http://www.megasoftware.net/mega6/) was used to construct phylogenetic trees with neighbor-joining (NJ) method (bootstrap analysis for 1000 repetitions). The evolutionary tree was visualized through EvolView online software [45].

### Gene structures and conserved motif analyses of *PtrXTHs*

The poplar genomic sequences were downloaded from the Phytozome12 database (https://phytozome.jgi.doe.gov/pz/portal.html). We used GSDS (http://gsds.cbi.pku.edu.cn/) online software to analyze the gene structure of the XTH family genes. MEME (http://meme-suite.org/tools/meme) online software was used to analyze the conserved motifs with default parameters, and the motifs were visualized with TBtools software [46].

### Cis-acting element analysis of *PtrXTHs*

The upstream 2000 bp sequences of the XTH genes were obtained from the Phytozome12 database The cis-elements in the all sequences were predicted through PlantCRAE (http://bioinformatics.psb.ugent.be/webtools/plantcare/html/), and the cis-elements were screened and visualized by TBtools software [46].

### Chromosomal locations, synteny and duplications analyses of *PtrXTHs*

The genomic data of the poplar was downloaded from phytozome12, and each gene was mapped to the corresponding chromosome position through the position information of the poplar XTH genes. We used the MCscan [47] to calculate the repetitive events of the *PtrXTHs* family genes among different species. Dual Synteny Plotter was drew to calculate the repetitive events of *PtrXTHs* between poplar and the other six species (*E. robusta*, *S. lycopersicum*, *G. max, Arabidopsis*, *Z. mays* and *O. sativa*) by TBtools software [46].

### Plant material and stress treatments

The experimental material used in this study was di-haploid *P. simonii* × *P. nigra and N. benthamiana*, which were cultured in the experimental forest of Northeast Forestry University, Harbin, China. The poplar seedlings were grown in 1/2 MS medium, and one-month-old seedlings were stressed with 0 and 150 mM NaCl for 0 and 12 h, respectively. The roots, stems and leaves at different time points were collected for RNA extraction. Meanwhile, the samples were sent to GENEWIZ Company for RNA-Seq with Illiumina platform [48]. The expression patterns of the *PtrXTH* genes in different tissues, and the DESeq2 [49] was used to screen the differentially expressed genes (DEGs) with absolute value of log_2_ (fold change) ≥1 and the adjusted *p*-value ≤0.05. The heatmap was drawn by TBtools [46]. In addition, we used RT-qPCR to verify the accuracy of DEGs screened by RNA-Seq. And the detailed information of RT-qPCR were referred to our previous studies [50]. All the samples were prepared with three biological replicates. All the primers were list in Supplementary Table 8.

## Supplementary Information

**Additional file 1: Supplementary Fig. S1.** Upset plot diagram of the poplar XTH genes throughout diverse species. The yellow color represents the number of genes that have collinearity between poplar and other species, the black circles connected by line segments represent genes that are shared by different species, and the black column represents the number of shared genes.

**Additional file 2: Supplementary Table 1.** Physicochemical characterization analysis of poplar XTH protein.

**Additional file 3: Supplementary Table 2.** Ten conserved motifs sequences found with MEME.

**Additional file 4: Supplementary Table 3.** The list of cis-regulatoty elements of *PtrXTH* gene promoter.

**Additional file 5: Supplementary Table 4.** Repetitive events of the *PtrXTHs.*

**Additional file 6: Supplementary Table 5.** The homologous relationships between the poplar XTH genes and the other species.

**Additional file 7: Supplementary Table 6.** Expression data of *PtrXTH* genes in three different tissues under salt and without salt stress.

**Additional file 8: Supplementary Table 7.** The fold-changes of differentially expressed *PtrXTH* genes under salt stress.

**Additional file 9: Supplementary Table 8.** The primers sequences used in this study.

## Data Availability

All data generated or analyzed during this study are included in this published article and its supplementary information files. The raw sequencing data used during the study have been deposited in NCBI SRA with the accession number SRP267437 (https://trace.ncbi.nlm.nih.gov/Traces/sra/?study=SRP267437).

## Abbreviations

XTH: Xyloglucan endotransglucosylase/hydrolase
XET: Xyloglucan endohydrolase
XEH: Xyloglucan endoglucosidase
MW: Molecular weight
DEGs: Differentially expressed genes
