# Supplementary figures and images for: Genome-wide identification and expression analysis of the xyloglucan endotransglucosylase/hydrolase gene family in poplar
Source: BMC Genomics. 2021 Nov 8;22:804. doi: 10.1186/s12864-021-08134-8 (PMC8576992; doi:10.1186/s12864-021-08134-8)

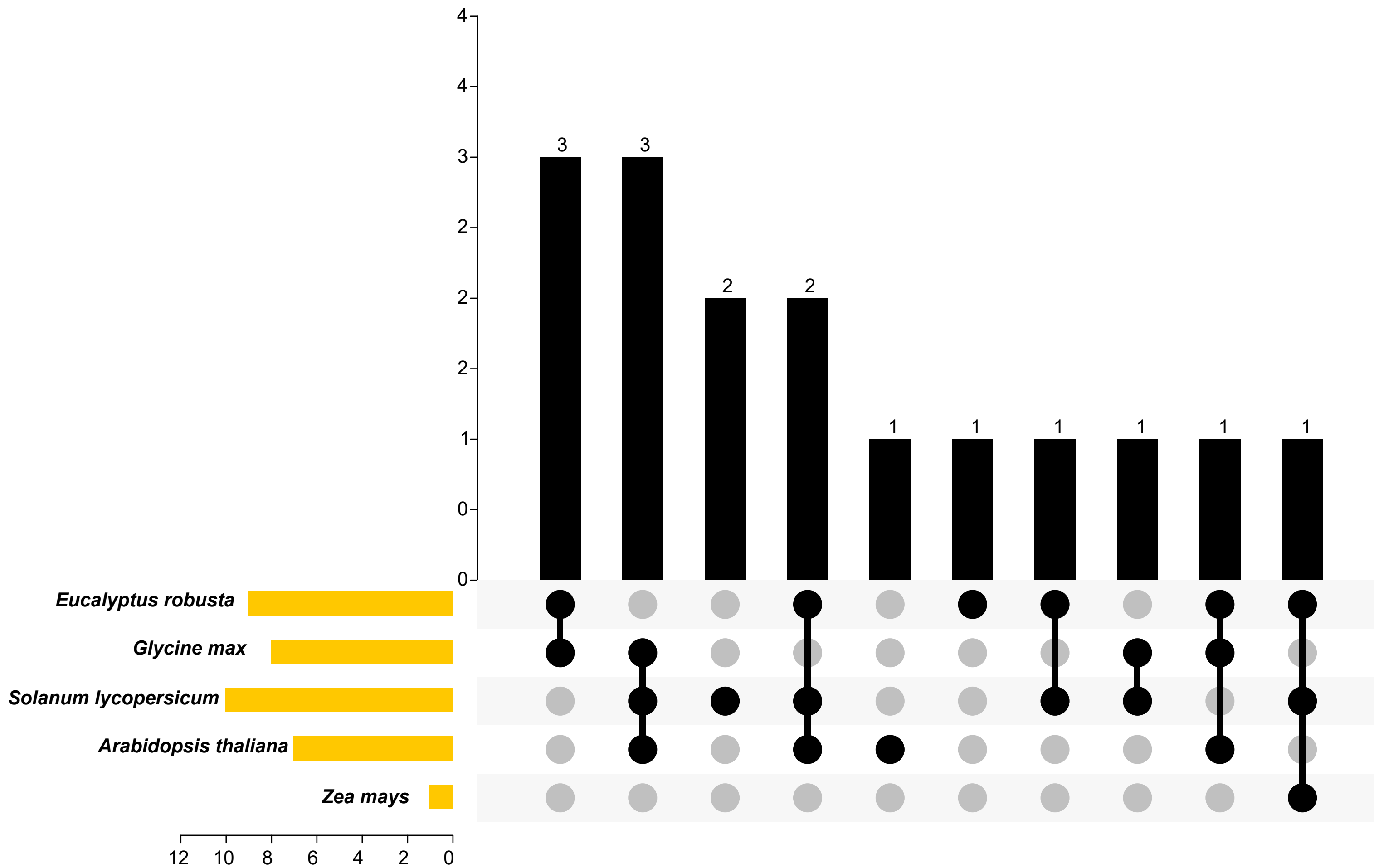

Supplement: Supplementary file 1 — Additional file 1: Supplementary Fig. S1. Upset plot diagram of the poplar XTH genes throughout diverse species. The yellow color represents the number of genes that have collinearity between poplar and other species, the black circles connected by line segments represent genes that are shared by different species, and the black column represents the number of shared genes. [file 12864_2021_8134_MOESM1_ESM.pdf]
